# *IGF1R* and *LOX* Modules Are Related to Antler Growth Rate Revealed by Integrated Analyses of Genomics and Transcriptomics

**DOI:** 10.3390/ani12121522

**Published:** 2022-06-11

**Authors:** Pengfei Hu, Zhen Wang, Jiping Li, Dongxu Wang, Yusu Wang, Quanmin Zhao, Chunyi Li

**Affiliations:** 1 Institute of Antler Science and Product Technology, Changchun Sci-Tech University, Changchun 130600, China; pfhoo@hotmail.com (P.H.); zhenw2019@126.com (Z.W.); ljpljp1994@163.com (J.L.); wdxcyc@hotmail.com (D.W.); jlauwang@163.com (Y.W.); 2 College of Traditional Chinese Medicine, Jilin Agricultural University, Changchun 130118, China; qm3657@163.com

**Keywords:** antler growth rate, genome-wide association analysis, weighted correlation network analysis, *IGF1R*

## Abstract

**Simple Summary:**

Previous studies on the growth rate of antlers are inconsistent, and few genes significantly related to growth traits have been obtained, which may be caused by the low-quality genome of sika deer or by the traditional genome-wide association analysis method being singly used. In this study, we conducted an integrated analysis of genome-wide association analysis and weighted gene co-expression network analysis using resequencing data identified in our previous analysis, which used antler weight and transcriptome sequencing data of faster- vs. slower-growing antlers of sika deer. The results show that a total of 49 genes related to antler growth rate were identified, and most of those genes were enriched in the *IGF1R* (*insulin-like growth factor 1 receptor*) and *LOX* (*lysyl oxidase*) modules. A gene regulation network of antler growth rate through the *IGF1R* pathway was constructed. We believe that our findings in the present study can provide further insight into revealing the molecular mechanism underlying the regulation of the tissue that can grow quickly without transforming into a tumor. Furthermore, the results of this study may be applied for increasing antler output for the deer industry.

**Abstract:**

Deer antlers are organs of bone and have an extremely rapid growth rate. Thus far, the molecular mechanism underlying rapid antler growth has not been properly elucidated, and key genes driving this growth rate have not been fully identified. In this study, based on the newly assembled high-quality sika deer genome, we conducted an integrated analysis of genome-wide association analysis (GWAS) and weighted gene co-expression network analysis (WGCNA) using genome resequencing data from our previous GWAS, with weight and transcriptome sequencing data of faster- vs. slower-growing antlers of sika deer. The expressions of key genes were verified using Fragments Per Kilobase of transcript per Million fragments mapped (FPKM) in different tissue zones of the antler growth center, different types of sika deer tissues and antler tissues collected from faster and slower growth rates. The results show that a total of 49 genes related to antler growth rate were identified, and most of those genes were enriched in the *IGF1R* and *LOX* modules. The gene regulation network of antler growth rate through the *IGF1R* pathway was constructed. In conclusion, the integration of GWAS and WGCNA analyses had great advantages in identifying regulatory genes of complex antler growth traits over using singular methods individually, and we believe that our findings in the present study can provide further insight into unveiling the mechanism underlying extraordinary fast antler growth rate in particular, as well as the regulatory mechanism of rapid tissue proliferation in general.

## 1. Introduction

Deer antlers are unique mammalian bony organs that are annually lost and regenerate, and they have an unprecedented growth rate during their growth period [1]. It has been recorded that antlers can grow up to 23 kg in 4 months [2]. The average growth rate of red deer is 1.8 cm/d, and that of sika deer is 1.2 cm/d [3]. Research has found that it is the rapid proliferation of antler tip cells that drive antlers to grow so rapidly [1,4,5,6]. Therefore, antlers can be used as an ideal model for studying extraordinary fast-growing tissue, revealing the molecular mechanism underlying the regulation of the tissue that can grow quickly without transforming into a tumor. Furthermore, the results of this study may be applied for increasing antler output for the deer industry in the future.

Previous studies have identified several genes related to antler growth rate, including *growth hormone 1* (*GH1*) [7,8], *melatonin receptor 1A* (*MTNR1A*) [9] and *androgen receptor* (*AR*) [8]. However, *insulin-like growth factor 1* (*IGF1*) was not identified through the methods used by the above mentioned studies, but *IGF1* is known as one of the most potent growth factors for stimulating antler growth, as there is a significant positive correlation between circulating *IGF1* levels (liver-sourced) and antler growth rate [10,11]. In our previous study, we conducted a full-length transcriptome analysis using antler growth center tissue (including reserve mesenchyme, RM), constructed a gene regulatory network using the identified 334 differentially expressed genes (DEGs) and found 14 genes that are related to antler growth rate [12]. None of these genes in our study, however, matched those reported in the previous studies. In another study, we identified 94 SNPs (single nucleotide polymorphisms) (*p* < 1 × 10^5^) using genome-wide association analysis (GWAS), but enrichment analysis on these SNPs failed to identify the pathways that are significantly involved in antler growth rate, which may be caused by the low-quality genome of sika deer or by the traditional GWAS method being singly used [13].

One of the biggest problems facing GWAS is the lack of detection ability for quantitative traits or complex traits controlled by multiple genes [14]. This is because complex biological process cannot be resolved through general association analysis [15], and the correlation between a single gene and a trait is relatively weak, thus often failing to reach a significant level after multiple tests and corrections in association analysis. Another reason is that a large number of pseudo-positive genes have been found in the ones that are categorized as “nominal” significant genes, which may affect the subsequent analysis. To solve these problems, we believe multistep methods should be used for allowing an integrated analysis to study complex attributes controlled by multiple genes [15,16], such as the integration of GWAS and the weighted gene co-expression network analysis (WGCNA) method [17,18].

The integration of GWAS and WGCNA provides great advantages in identifying regulatory genes of complex biological processes over using singular methods individually. If the co-expression module in WGCNA analysis is related to a phenotype, it means that the gene set of the module is involved in the regulation of a phenotype, and the hub regulatory genes in the module are often related to the phenotype in GWAS. Therefore, mutual verification provides more accurate clues for the subsequent analyses. In addition, using the mutual regulatory relationship between genes, the functions of unknown genes can be predicted from known genes, and new functional genes can be identified.

In this study, based on the newly assembled high-quality sika deer genome [19], we conducted an integrated analysis of GWAS and WGCNA using genome resequencing data from our previous GWAS, which used antler weight and transcriptome sequencing data of faster- vs. slower-growing antlers of sika deer [12,13]. For the first step, GWAS was used to identify the genes that are related to antler growth with lower screening thresholds. For the second step, transcriptome data from the faster- vs. slower-growing antlers were used to verify the candidate genes using WGCNA. Through the approach of the integration of WGCNA and GWAS, key genes with biological significance and in the mutation sites were identified. Finally, the expressions of key genes were verified using Fragments Per Kilobase of transcript per Million fragments mapped (FPKM) in different tissue zones of the antler growth center, different types of sika deer tissues and antler tissues collected from faster and slower growth rates. Overall, our study improved the detection accuracy and sensitivity of key genes regulating antler growth rate in sika deer that could have not been found using singular analysis methods. We believe that our findings are of great importance for revealing the mechanism underlying extraordinarily fast antler growth rate in particular, as well as the regulatory mechanism of rapid tissue proliferation in general.

## 2. Materials and Methods

### 2.1. Data Collection and Preparation

The high-quality sika deer genome was obtained from the National Genomics Data Center (accession number GWHANOY00000000, https://bigd.big.ac.cn/gwh, accessed on 28 September 2021). The antler-growth-rate-related genome re-sequencing data of 100 sika deer individuals with different antler growth rates (Genbank accession number: PRJNA541418) were used for GWAS. The phenotypic information of the 100 individuals used for GWAS was recorded in the published article [12]. They were the same age and from the same sika deer population, raised under the same conditions and fed the same diet. According to the weight of the antlers within the same growth period (the time from shedding to harvesting), we divided the 100 individuals into two groups: faster- and slower-growing groups (at 75 days of growth, antlers weighed 5.92 ± 0.15 kg in the faster-growing group and 2.56 ± 0.04 kg in the slower-growing group, *p* < 0.01).

The expression levels of antler-growth-rate-related transcripts for the faster-growing antlers compared to the slower-growing antlers were obtained from publicly available data (Genbank accession number: PRJNA470791), from three faster-growing antlers from three individual sika deer (deer ID: 137, 1162 and 395; antlers weighed 7.98, 5.79 and 6.26 kg at 75 days of growth, respectively) and from three slower-growing antlers from three individual sika deer (deer ID: 003, 1729 and 518; antlers weighed 2.78, 2.84 and 2.95 kg at 75 days of growth, respectively). Their phenotypic information is recorded in a published article [12], and for each antler, the skin (D), reserve mesenchymal and pre-cartilage (RP), transition zone and cartilage (TC) were sequenced. In total, there were nine antler samples in each group. The gene expression data used for the verification of transcripts of genes regulating antler growth rates were downloaded from a publicly available database (Genbank accession number: PRJNA408029 and PRJNA438286).

### 2.2. Identification of Antler-Growth-Rate-Related SNPs Using GWAS

The genome re-sequencing data were re-analyzed using BWA software (parameters: aln-e10-l32-i15-q10). The duplication of mapping results was removed, and total SNPs were identified using SAMTOOLS software (mpileup-m2-F0.002-d1000). Polymorphic loci in the sika deer population were evaluated using the Bayesian model, and then the SNPs were filtered using the following threshold: the SNP site read that the supported vcf file was more than 2, the missing ratio of SNP was less than 10% and the minimum allele frequency was more than 5%. The finally obtained SNPs were annotated using ANNOVAR software.

The identified SNPs were used for population principal components analysis (PCA) using GCTA software, and linkage disequilibrium (LD) analyses using Haploview software were performed with the following parameters: maximum distance of 500 kb and a minimum MAF of 0.05. Before GWAS, one abnormal individual was removed based on the normal square distribution of the antler weights of 100 individuals. GWAS was carried out using GEMMA software (http://www.xzlab.org/software.html, accessed on 15 October 2021), and antler-growth-rate-related SNPs were identified according to the level of significant *p* value of association, calculated using a mixed linear model (MLM). Based on the analysis of the attenuation distance of linkage disequilibrium, the functional annotation of related genes in a certain region upstream and downstream of the physical location of the significant SNP locus was carried out.

### 2.3. Identification of Transcripts Related to Antler Growth Rate Using the Integration of GWAS and WGCNA

The antler-expressed transcripts (AETs) that contained antler-growth-rate-related SNPs from GWAS, together with the differentially expressed transcripts (DETs) with or without the SNP sites, were combined to perform WGCNA. The missing or low-expression transcripts were firstly filtered out, and then a sample cluster tree was used to determine whether there was an outlier sample. The Pearson coefficients between any two transcripts in the filtered dataset containing AETs and DETs were calculated, and the N-power of the Pearson coefficients was taken to establish the connection between transcripts in the network that fit the scale-free network distribution. The correlation network between transcripts was constructed using the power value, and the cluster tree and corresponding module were visualized. The mean value of the correlation between the trait and the expression level of each transcript in each module was taken as the significant degree of the trait association in the module, and the most significant module was considered to be the most relevant to antler growth rate.

### 2.4. Gene Screening in the Modules and the Construction of Gene Interaction Networks

Genes in the modules with a high correlation with antler growth rate were further screened, and a GO and KEGG enrichment analysis of the identified genes was carried out using the online tool David 6.8 (https://david.ncifcrf.gov/, accessed on 29 October 2021), with a *p* value < 0.05 as the criterium of significant enrichment. The interaction relationship of the genes was analyzed using the STRING database (https://string-db.org version 11.0, accessed on 29 October 2021), taking cattle as the reference species, and the confidence value was set to 0.9. The gene interaction pairs were imported into Cytoscape 3.6.0 software, and then the sub-network modules were analyzed using Cluste One plug-in. A functional enrichment analysis of genes in the sub-network modules with significant interaction values was carried out. The CytoHubba plug-in from Cytoscape 3.6.0 software was used to explore multiple genes from the interaction network, based on eleven topological algorithms, including Degree, Edge Percolated Component (EPC), Maximum Neighborhood Component (MNC), Density of Maximum Neighborhood Component (DMNC), Maximal Clique Centrality (MCC), centrality based on the shortest path as a bottleneck (BN), EcCentricity, Closeness, Radiality, Betweenness and Stress. Genes with node degree of greater than 10 were considered to be closely related to antler growth rate. A regulatory network of genes for antler growth rate was constructed based on the above results.

### 2.5. Verification of Transcripts of Genes Regulating Antler Growth Rate

The expression levels of the transcripts of genes in different antler growth zones (Reserve mesenchyme, Pre-cartilage, Transition, Cartilage and Mineralized Cartilage), different tissues (heart, liver, spleen, lung, muscle, skin, rumen, esophagus, cecum and brain) and antlers with faster and slower growth rates were analyzed using published data. The sequencing reads of each sample were mapped to the reference sika deer genome with Bowtie2 v2.0.5 (—no-mixed—no-discordant—gbar 1000—end-to-end-k200), and a statistical analysis of the read count was conducted using the mapping results by RSEM v1.3.0. The Fragments Per Kilobase of transcript per Million fragments mapped (FPKM) results of each sample are presented as means ± SE and are visualized using HemI 1.0 software.

## 3. Results

### 3.1. Identified Genes Related to Antler Growth Rate Using GWAS

**Figure 1:**
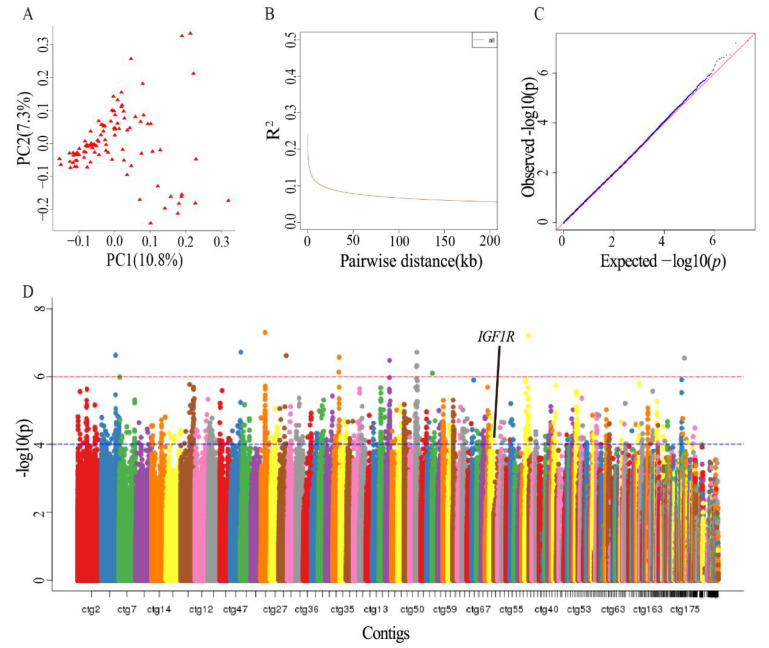
The GWAS of SNPs related to antler growth rate: (**A**) The analyzed population was a natural population and did not form significant genetic differentiation. (**B**) A value of 20 kb was set as the LD value of GWAS. (**C**) Most of the observed and expected values of the *p*-value are the same, which indicates that the analysis model is reasonable. The point above the diagonal in the upper right corner is a significant site, but it does not exceed the expected value. (**D**) When the *p* value ≥ 1 × 10^−6^ (shown by the red dotted line), only 13 associated SNPs were obtained; when *p* value ≥ 1 × 10^−4^ (shown by the blue dotted line), 1182 associated SNPs were obtained.

A high-quality sika deer genome was used in this study, and this genome was considered as a deer reference genome. The genome re-sequencing data of 100 sika deer with fast and slow antler growth rates were mapped to the reference genome, and from this mapping, a total of 39,788,029 SNPs were identified. After filtration, 11,351,008 non-redundant SNPs were obtained. Principal component analysis (PCA) performed on the identified SNPs showed that all individuals were not clustered into distinct subgroups, indicating that the analyzed population is a natural population and does not form significant genetic differentiation (Figure 1A). The linkage disequilibrium (LD) analysis shows that the LD value decreased rapidly, indicating that the sika deer population had a low recombination rate, a low domestication degree and a weak selection intensity. The threshold set in this study was such that SNPs were linked in the range LD coefficient (R2) of greater than 0.1, and the horizontal ordinate corresponding to the value was about 16 kb (Figure 1B). The distance was expanded, and 20 kb was set as the LD value for GWAS.

In GWAS, the population’s genetic structure was regarded as a fixed effect, and individual relationships were regarded as a random effect, in order to correct the effects of population structure and individual relationships. The analysis model was reasonable through the evaluation of a QQ plot map, for which the observed and expected *p* values were mostly consistent (Figure 1C). The points in the upper right corner above the diagonal are the significant sites, but most of these sites did not exceed the expected value significantly, which further shows that rapid antler growth was a complex attribute controlled by a large number of genes. When the *p* value ≥ 1 × 10^−6^ (Figure 1D, above the red dotted line), only 13 SNPs were identified. When the *p* value ≥ 1 × 10^−4^ (Figure 1D, above the blue dotted line), 1182 SNPs were identified. Functional annotation was carried out for the relevant genes in the region of 20 kb upstream and downstream of the physical location of the 1182 SNP sites, and a total of 329 genes were annotated, comprising 631 SNP sites. Among them, *IGF1R* had an A to G mutation at the 11,323,595th site of the 39th count in the sika deer genome.

### 3.2. Identified Genes That Are Related to Antler Growth Rate Using the Integration of GWAS and WGCNA

**Figure 2:**
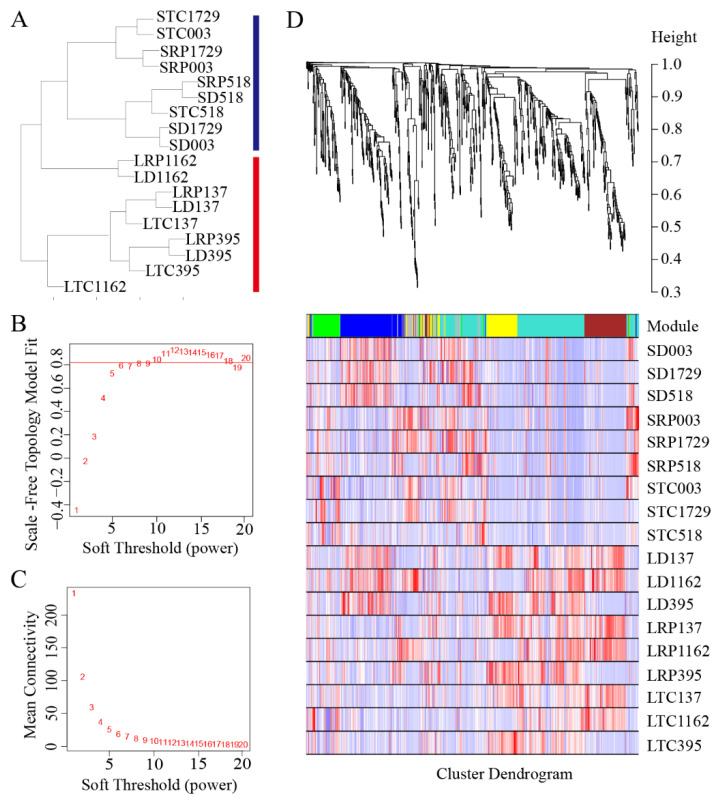
The WGCNA analysis of genes related to antler growth rate: (**A**) Based on 1564 transcripts, a hierarchical cluster tree was constructed. The red line indicates the fast-growing velvet antler sample, and the blue line indicates the slow-growing velvet antler sample. (**B**,**C**) The power curve of the scale-free network shows that the optimal soft threshold is six. (**D**) According to the correlation of gene expression, a cluster tree was constructed, which was divided into six modules.

**Figure 3:**
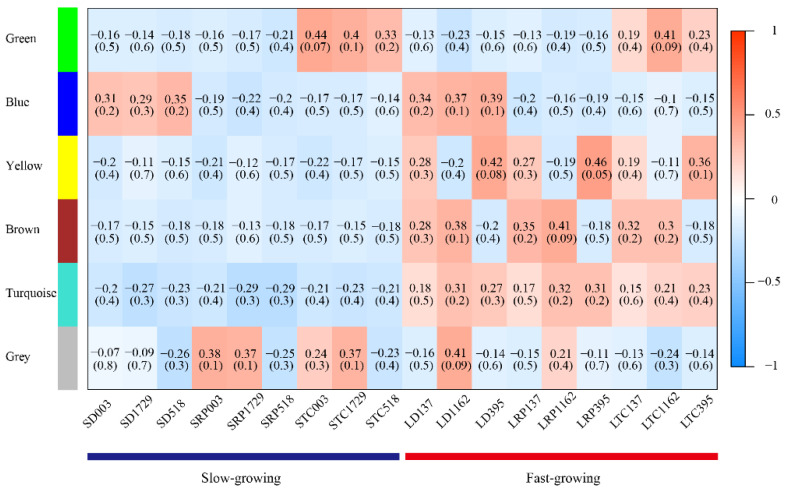
Relationship between module and sample. Each row represents a module, and each column represents the antler growth center tissue of an individual. The correlation increases with a redder color. The correlation analysis among samples and modules shows that yellow, brown and turquoise modules were closely related to antler growth rate.

Of the 329 genes annotated, 177 were found to be expressed in the antler growth center. The 933 transcripts of the 177 genes and 645 DEGs in the growth centers of faster vs. slower growing antlers were combined to form 1564 transcripts. An analysis of these 1564 transcripts using WGCNA shows that samples from the slower-growing antlers were clustered into a branch, whereas samples from the faster-growing antlers were clustered into another (Figure 2A). Based on the optimum soft threshold power calculated using the power value curve of the scale-free network (Figure 2B,C), the clustering tree of the correlation of gene expression levels was constructed, and six modules were identified (Figure 2D), with 781 transcripts (155 in blue module, 113 in brown module, 71 in green module, 65 in grey module, 272 in turquoise module and 105 in yellow module) being included. The expression level of each transcript in the six modules is shown in Figure 2D, and the relationships between modules and samples were calculated. The results show that the yellow, brown and turquoise modules were closely related to antler growth rate (Figure 3). The transcripts in these three modules were annotated, and a total of 277 genes were then identified to be related to antler growth rate.

### 3.3. Functional Enrichment and Interaction Network of Genes Related to Antler Growth Rate

Many GO terms and KEGG pathways were identified through the functional enrichment of 277 genes and have failed to be shown in previous studies using a single analysis method (Table S1), such as the PI3K-Akt signaling pathway that was significantly enriched (*p* = 2.47 × 10^7^), indicating that we have effectively isolated the genes related to antler growth rate from many candidate genes using WGCNA combined with GWAS. We focused on 49 genes in the biological functions and signaling pathways that stimulate cell proliferation (Table 1) for further investigation, and these genes were considered to be genes for regulating antler growth rate. These 49 genes had 2663 transcripts, and the expression levels of each transcript in the antler growth center is shown in Table S2. Among these genes, 28 were highly expressed in the antler with an FPKM value of larger than 50 (Table S3).

**Figure 4:**
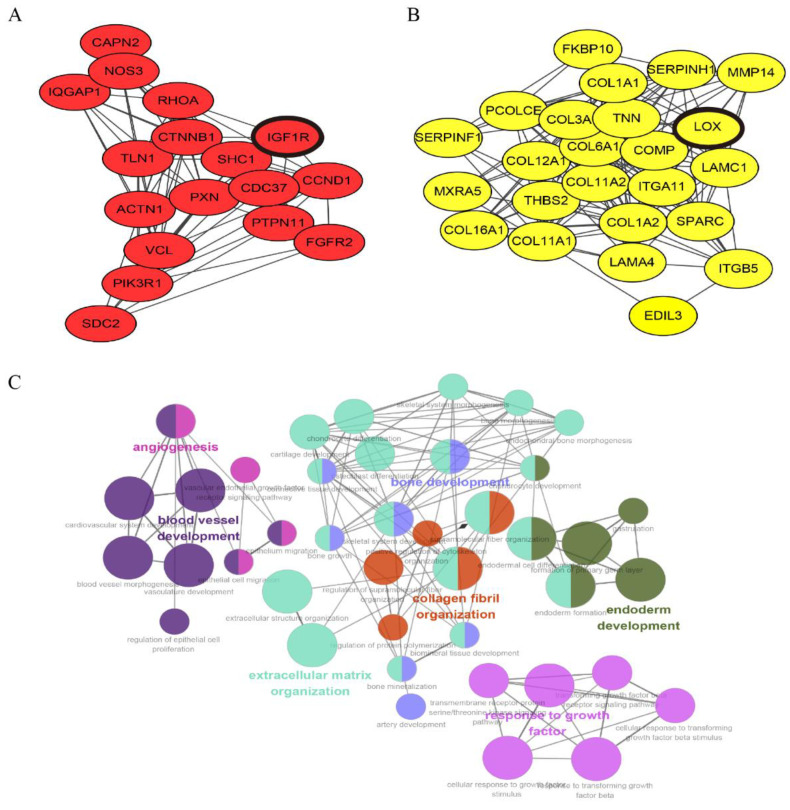
Gene interaction network related to antler growth rate. The interaction network of 277 antler-growth-rate-related genes identifies two sub modules with most of the 49 minor genes, namely the *IGF1R* module (**A**) and *LOX* module (**B**), and they were relatively close to each other in the whole interaction network. Enrichment networks of 277 genes were constructed using Cytoscape plug-in Cluego (**C**), and each node is a representative enrichment pathway. The connection of nodes represents the number of genes shared between the pathways, and the color indicates the classification of the node enrichment. The results show that these genes have significant interactions in biological processes such as bone development, extracellular matrix organization and collagen fibril organization.

The interaction network of 277 antler-growth-rate-related genes was constructed. In the network, 32 genes from 49 genes had an interaction degree value of larger than 10 (Table S3), indicating that these genes may play key roles in regulating antler growth rate. There were two sub modules that contained most of the 49 genes, namely the *IGF1R* module and *LOX* module (Figure 4A,B) (Table S4), and they were relatively close to each other in the whole interaction network, indicating that they are closely related in function. Enrichment networks of 277 genes were constructed (Figure 4C), and the results show that these genes have significant interactions in biological processes such as bone development, extracellular matrix organization and collagen fibril organization.

### 3.4. Expressions of Different Transcripts of IGF1 and IGF1R in Sika Deer

Previous studies have shown that the *IGF1* gene plays a key role in regulating antler growth, so we further analyzed the expression characteristics of different transcripts of *IGF1* and *IGF1R* (key minor gene) in sika deer. Eleven transcripts of the *IGF1* gene were identified through transcriptome analysis of sika deer tissues, and the length of the coding region of the *IGF1* gene was 414–567 bp, which consisted of 5 exons and 9 isoforms. The sequences of sika deer *IGF1* isoform 5 and human *IGF1* isoform 4 were the closest (94.12%) and had nine different amino acids. Although only one transcript of *IGF1R* was identified through the transcriptome analysis of sika deer tissues, the length of the coding region of the *IGF1R* gene was 4053 bp, which consisted of 21 exons and 1 isoform. The sequences of sika deer *IGF1R* isoform and human *IGF1R* isoform 1 were the closest (95.39%).

**Figure 5:**
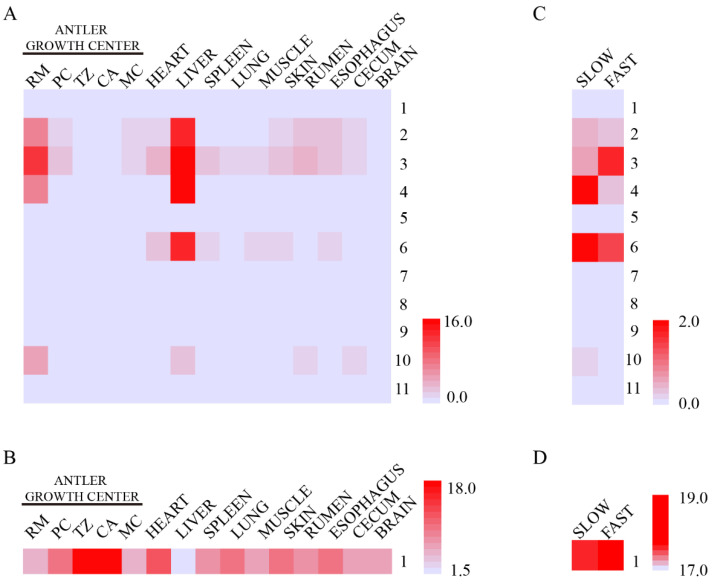
Transcript expression of *IGF1* and *IGF1R* in antlers and different tissues of sika deer: (**A**) Expression of 11 transcripts of *IGF1* gene in different tissues of sika deer. In addition to the liver (the main source of *IGF1*), the highest expression of *IGF1* was found in the RM layer of the antler growth center. (**B**) Expression of the transcript of the *IGF1R* gene in different tissues of sika deer. The transcript expression level of *IGF1R* in the antler growth center was higher than in other tissues and organs. (**C**) Expression of 11 transcripts of the *IGF1* gene in antlers with different growth rates. The expression levels of transcripts 3 and 4 of the *IGF1* gene were significantly different between the faster and slower-growing antlers. (**D**) Expression of the transcript of the *IGF1R* gene in antlers with different growth rates. There were no different expression levels of the transcripts of the *IGF1R* gene found between faster and slower-growing antlers.

Expression levels of *IGF1* in both the liver and antler RM zone were significantly higher than those of other tissue types (Figure 5A), suggesting that, in addition to the endocrine source of *IGF1* (liver), the proliferation of RM cells may also be stimulated through autocrine *IGF1* (antler). Furthermore, the transcript expression level of *IGF1R* in the antler growth center was higher than in other tissues and organs (Figure 5B). With the stimulation of *IGF1* from both the endocrine and autocrine and the highest expression of *IGF1R* in the antler growth center, antler growth can certainly achieve an exceptionally high speed. Expression levels of transcripts 3 and 4 of the *IGF1* gene were significantly different between the faster and slower-growing antlers (Figure 5C), suggesting that the different *IGF1* transcripts may play different roles in antler growth rate, which are worthy of further study. There were no different expression levels of the transcripts of the *IGF1R* gene between faster and slower-growing antlers (Figure 5D).

## 4. Discussion

It is generally agreed that a complex trait is not completely controlled by a single gene, and multiple genes are normally involved [20]. We believe that numerous genes related with antler growth rate have not been identified yet. Therefore, in this study the whole genome re-sequencing data and transcriptome data of sika deer with different antler growth rates were re-analyzed using the integration of GWAS and WGCNA. Consequently, we identified a series of new SNP sites and more antler-growth-rate-related genes, and some of these SNPs and genes have not been reported thus. *IGF1R* identified in this analysis further confirmed the previous findings that *IGF1/IGF1R* are the key genes in regulating antler growth rate. We further found that many genes related to antler growth rate were enriched in the PI3K/Akt signal pathway, and thus, this pathway may also play a key role in regulating antler growth. The PI3K/Akt signal pathway was identified in the antler growth center in our previous studies [21,22], and interestingly, this pathway is downstream of *IGF1/IGF1R* for driving cells to divide [23]. In addition, other *IGF1/IGF1R*-related genes and signal pathways were significantly enriched, and they may also play an important role in the regulation of antler growth rate.

Multiple genes, especially the *IGF1R* gene identified in this study, were previously considered to be key genes in regulating antler growth rate. It is known that type 1 IGF receptors are richly localized in the antler growth center [24], so antlers are major target organs of *IGF1*. Further studies have shown that *IGF1*, like in the other tissues and organs, also plays a regulatory role in antler growth by binding to *IGF1R* [25]. In vitro experiments have shown that *IGF1* has a mitogenic effects on cells from the pedicle periosteum and antler RM in a dose-dependent manner [26,27,28]. Therefore, *IGF1* is an important mitogen of cells in the antler growth center. Our studies have shown that the expression level of the *IGF1* gene in the antler growth center was higher than that in other organs, except for the liver (the major endocrine source of *IGF1*), and the expression level of the *IGF1R* gene in the growth center of antlers was higher than in any other organs/tissues. We believe that, in the stage of rapid antler growth, in addition to the endocrine source of *IGF1* (liver), the paracrine/autocrine source of *IGF1*(RM cells) can also stimulate antler growth. Therefore, the dual source of *IGF1* and the high expression of *IGF1R* in the antler growth center may constitute a very powerful regulation network for rapid antler growth.

It has been reported that *IGF1* promotes the proliferation of intestinal epithelial cells in obese people via PI3K/Akt rather than via ERK signaling [29]. The proliferation of myoblasts has been reported to be mediated by changes in the PI3K/Akt and MAPK pathways via *IGF1R* [30]. *IGF1* promotes the proliferation of retinal precursor cells by binding to *IGF1R*, and it stimulates phosphorylation of the node protein in the PI3K/Akt and MAPK/ERK pathways. Further experiments have shown that blocking the PI3K/Akt and MAPK/ERK pathways inhibit the *IGF1*-stimulated proliferation of retinal precursor cells [31]. A growth inhibition experiment using MDA-MB-231 cells has shown that the inhibition of *IGF1R*, *PI3K*, *mTORC* or mitogen extracellular signal-regulated kinase (MEK) can induce the apoptosis of MDA-MB-231 cells and arrest the cell cycle at the G1 phase [32]. All of this evidence demonstrates that the *IGF1/PI3K/Akt* axis is a very important pathway for the regulation of cell proliferation and the cell cycle. Based on these findings, we speculate that the PI3K/Akt signaling pathways mediated by *IGF1/IGF1R* may regulate the growth rate of antlers and stimulate antler growth to a phenomenal speed.

**Figure 6:**
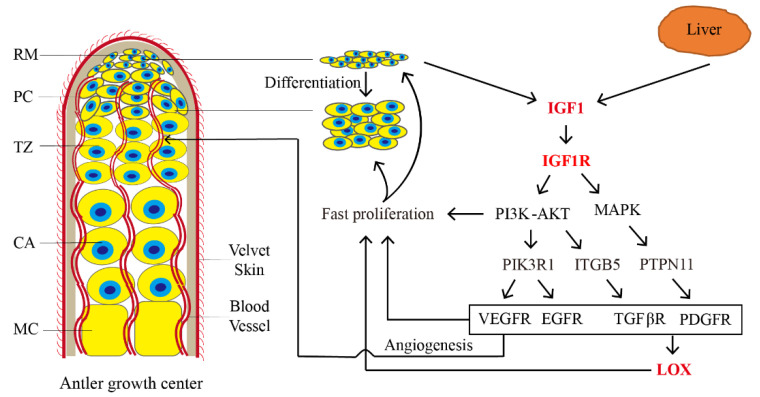
Gene regulation network of antler growth rate stimulated by *IGF1/IGF1R*. In the network, after activating *IGF1/IGF1R*, genes in PI3K/AKT and MAPK signaling pathways may be up-regulated, and expression of *PIK3R1, ITGB5* and *PTPN11* may promote VEGFR, EGFR, TGFƥR and PDGFR signal pathways to realize fast proliferation of RM cells. Genes in VEGFR signal pathways may also promote angiogenesis in antlers to ensure the nutrition supply for rapid growth. Activation of genes in TGFƥR and PDGFR signal pathways may induce expression of *LOX*, which may mediate ECM remodeling, activate cell signaling and gene transcription and further promote rapid antler growth. RM, reserve mesenchyme; PC, pre-cartilage; TZ, transition; CA, cartilage; MC, mineralized cartilage.

Multiple genes identified in this study may be regulated by *IGF1R*, such as *VEGF* and *EGFR*. In SCC9 cells, *IGF1/IGF1R* can stimulate the PI3K/Akt and ERK/MAPK pathways, via stimulating *HIF1A* expression and *VEGF* secretion, respectively [33]. In this study, we also found that most of the 49 genes were significantly enriched in the VEGFR, EGFR, INSR, PDGFR, TGFβR and MAPK pathways, which supports previous reports that suggest that many growth factors and receptors in these pathways are expressed in the antler growth center and play important roles in regulating antler growth [34,35,36,37]. Based on the above information and our results, an *IGF1R* regulation network involving genes and signal pathways related to antler growth rate was constructed (Figure 6). In the network, after the activation of *IGF1/IGF1R*, genes in the PI3K/AKT and MAPK signaling pathway may be up-regulated, and the expression of *PIK3R1*, *ITGB5* and *PTPN11* may activate the VEGFR, EGFR, TGFƥR and PDGFR signaling pathways to stimulate fast the proliferation and differentiation of RM cells. Genes in the VEGFR signaling pathways may also participate in the process by promoting angiogenesis in antlers to ensure ample energy and nutrition available for sustaining rapid growth. The activation of genes in the TGFƥR and PDGFR signaling pathways may induce the expression of *LOX*, which may mediate extracellular matrix (ECM) remodeling, activate cell signaling and gene transcription and further promote rapid antler growth. However, the detailed molecular mechanism regarding how *IGF1R*, together with other genes and signaling pathways, sustains an unprecedented growth rate for antlers requires further study. We believe that these findings provide important clues for revealing the mechanisms underlying antler growth rate at the molecular level. It can also be useful for future studies, particularly for the deer industry and for medical applications.

## 5. Conclusions

In summary, we identified 49 genes related to the antler growth rate of sika deer using the integration of GWAS and WGCNA, and the gene interaction network revealed that most of the 49 genes were enriched in *IGF1R* modules and *LOX* modules. A gene regulation network of antler growth rate activated by *IGF1/IGF1R* was constructed. We believe that our findings in the present study are of great importance for revealing the mechanism underlying extraordinarily fast antler growth in particular, as well as the regulatory mechanism of rapid tissue proliferation in general.

## Figures and Tables

**Table 1:** The 49 genes in the biological functions and signaling pathways that are related to antler growth rate.

Term	Genes
vascular endothelial growth factor receptor signaling pathway	*NRP2, DOCK1, NRP1, BAIAP2, RHOA, PIK3R1, PXN, NCKAP1*
epidermal growth factor receptor signaling pathway	*SHC1, PIK3R1, IQGAP1, PXN, PTPN11*
ERBB2 (HER2) signaling pathway	*UBC, SHC1, PIK3R1, CDC37*
cellular response to epidermal growth factor stimulus	*MCM7, COL1A1, SNAI2, IQGAP1*
insulin receptor signaling pathway	*IGF1R, BAIAP2, SHC1, APPL1, PIK3R1*
platelet-derived growth factor receptor signaling pathway	*NRP1, IQGAP1, PTPN11*
transforming growth factor beta receptor signaling pathway	*COL3A1, RHOA, COL1A2, UBC, ITGB5, ENG, PXN*
negative regulation of transforming growth factor beta receptor signaling pathway	*GLG1, HTRA1, UBC, ENG*
ephrin receptor (subfamily of RTKs) signaling pathway	*ARPC1A, ARPC1B, AP2A2, ARPC2, RHOA, SDC2, PTPN11*
activation of MAPK activity	*CD81, UBC, SHC1, LPAR1, PTPN11*
positive regulation of mesenchymal cell proliferation	*FGFR2, PRRX1, CTNNB1*
PI3K-Akt signaling pathway	*HSP90AB1, FGFR2, COL3A1, ITGA11, ITGB5, LPAR1, CDC37, IGF1R, CCND1, HSP90B1, LAMA4, COMP, COL1A2, YWHAQ, COL6A1, TNN, NOS3, GNB4, COL1A1, LAMC1, COL11A2, THBS2, COL11A1, PIK3R1*

Through the integration analysis of GWAS and WGCNA, 277 genes were obtained which were highly related to the growth rate of antlers. The enrichment analysis of 277 genes shows that 49 genes are involved in the biological function of promoting cell proliferation. Therefore, we speculated that these 49 genes are key genes regulating the rapid growth of antlers.

## Data Availability

The raw data are available from the SRA (http://www.ncbi.nlm.nih.gov/sra/, accessed on 28 September 2021) data repository (accession number: PRJNA541418, PRJNA408029, PRJNA438286 and PRJNA470791) and the National Genomics Data Center (https://bigd.big.ac.cn/gwh, accessed on 28 September 2021) (accession number: GWHANOY00000000).

## Supplementary Materials

The following supporting information can be downloaded at: https://www.mdpi.com/article/10.3390/ani12121522/s1, Table S1: The functional enrichment of 277 genes related to antler growth rate; Table S2: The expression of 2663 transcripts from 49 genes in antlers; Table S3: The 28 genes that are highly expressed with FPKM of larger than 50; Table S4: Sub-module analysis of 277 antler-growth-rate-related genes.
